# The Mixed Board Bill*A provisional Practice Act for consideration by legislative committees and county societies.

**Published:** 1906-04

**Authors:** 


### The Mixed Board Bill.*

A BILL

TO BE ENTITLED

*An Act to control the practice of the healing art; create a board of examiners; provide for the licensing of practitioners and their proper registration, and for the revocation of licenses for flagrant offenses, and to fix suitable penalties for illegal practice.*

SECTION 1. Be it enacted by the Legislature of the State of Texas that a board, to be known as the Board of Medical Examiners for the State of Texas, is hereby established, the terms of office of whose members shall be for two years or until their successors shall be appointed and qualified.

SEC. 2. That said board shall consist of men learned in the healing art and in active practice and legalized practitioners in the State of Texas, who shall have resided and legally practiced the healing art in this State for more than five years prior to their appointment.

The board shall be appointed by the Governor of this State on the first day of September following his inauguration, and the members of said board shall be selected in the following manner: The representatives of each school of medicine or healing art in this State, who desire a member of such school on said board, shall, on or before the first day of August after the inauguration of each Governor, file with the Secretary of State of this State a copy of the by-laws, rules and regulations of its State organization, together with the names and addresses of each known legal and authorized practitioner under the laws of Texas belonging to such school, which report shall be verified by the affidavits of the president and secretary of such State organization. There shall be selected by the said Governor one member of said board for every five hundred members and fractional part thereof over two hundred and fifty of each school, as shown by the report filed in the office of the said Secretary of State; provided, that all schools of medicine or healing art, which have complied with all the foregoing conditions, shall have at least one representative on said board, and provided further, that no school of medicine or healing art which does not maintain a separate State organization shall be entitled to any representation on said board.

The State organization of each separate school shall, on or before the

*A provisional Practice Act for consideration by legislative committees and county societies.

first day of August after the inauguration of the Governor of this State, recommend to the said Governor a list of names of twice the number to be appointed from such school, and the appointments shall be made by the Governor from said lists, unless the Governor shall consider any of the persons so recommended on either of said lists unsuitable, and, in such case, he shall communicate the fact to the presiding officer presenting the nominations, and such society shall, within sixty days thereafter, make other recommendations. Vacancies occurring in said boards shall be filled from the lists already in the hands of the Governor. Unless the said second lists of recommendations herein provided for are not submitted within sixty days after giving such notice by the Governor, then he shall at his own discretion fill the vacancies on the said board.

SEC. 3. All osteopathic practitioners in this State, who were in good standing in the Texas State Osteopathic Association on January 1, 1905, and who shall present to the Board of Medical Examiners for the State of Texas within sixty days after the passage of this act, satisfactory evidence of that fact, and that they are graduates of bona fide, reputable osteopathic colleges, shall be granted by said board a certificate, and upon registration of the same in the district clerk's office, as provided in this act, they shall be entitled to practice osteopathy in this State, but such certificate shall not permit them to administer drugs or perform surgical operations.

SEC. 3. The members of such board shall qualify by taking the oath of office before a notary public or other officer empowered to administer oaths in the county in which they shall respectively reside. At the first meeting of said board after each biennial appointment the board shall elect a president, vice-president and secretary-treasurer. Six members shall constitute a quorum. Regular meetings shall be held at least twice a year at such times and places as shall be deemed most convenient for applicants. Due notice of such meetings shall be given by publication in such papers as may be selected by the board. Special meetings may be held upon a call of three members of the board. The board may prescribe rules, regulations and by-laws for its own proceedings and government for the examination of applicants for the practice of the healing art and obstetrics. Said board shall have power to administer oaths for all purposes required in the discharge of its duties, and to adopt a seal to be affixed to all its official documents. No member of the board shall be a stockholder, a member of the faculty or board of trustees of any medical school.

SEC. 5. The board of examiners shall keep a record of its proceedings in a book kept for that purpose, showing name, age, place and duration of residence of each applicant, the time spent in medical study in respective medical schools, and the year and school from which degrees were granted; said register shall also show whether applicants were rejected or licensed, and shall be prima facie evidence of all matters contained therein. The secretary of the board shall on March 1st of each year transmit an official copy of said register to the Secretary of State for permanent record.

SEC. 6. It shall be unlawful for any one to practice the healing art in any of its branches upon a human being within the limits of this State who has not registered in the district clerk's office of the county in which he resides his authority for so practicing, as herein prescribed, together with his age, postoffice address, place of birth, school of practice to which he professes to belong, subscribed and verified by oath, which if wilfully false shall subject the applicant to conviction and punishment for perjury. The fact of such oath and record shall be endorsed by the district clerk upon the certificate. The holder of the certificate must have the same recorded upon each change of residence to another county, and the absence of such record shall be prima facie evidence of the want of possession of such a certificate.

SEC. 7. It is hereby made the duty of the district clerk of each county in this State to purchase a book of suitable size, to be known as the "Medical Register" of such county, and to set apart one full page for the registration of each physician, and to record in the same the name and record of each practitioner who presents a certificate from the State Board of Examiners, issued under this act. When any physician shall die, or remove from the county, or have his license revoked, it shall be the duty of the clerk to make a note of the facts at the bottom of the page as closing the record. On the first day of January in each year such clerk shall, on request of the board, certify to the office of the State Board of Medical Examiners a correct list of the physicians then registered in the county, together with such other information as said board may require. The clerk shall receive the sum of one dollar from each physician so registered, which shall be his full compensation for all duties required under this act.

SEC. 8. From and after the passage of this act authority to practice any branch of the healing art in this State shall be a certificate from the State Board of Medical Examiners, registered as herein provided in the county in which the holder resides. Within one year after the passage of this act all legalized practitioners of the healing art in this State, who have not received licenses from a State medical examining board shall present to the Board of Medical Examiners for the State of Texas legally certified transcripts of such legality, or affidavits from persons competent to establish such legality, and shall receive from said board verification licenses, which shall be recorded in the district clerk's office in the county in which licentiate may reside. Such verification licenses shall be issued for a fee of fifty cents to practitioners recognized by the laws of this State as legalized prior to the passage of this act, provided that all whose claims to State licenses rest upon diplomas from medical colleges recorded from January 1, 1891, to July 9, 1901, present to the State Board of Medical Examiners satisfactory evidence that their diplomas were issued by bona fide medical colleges of reputable standing before they are entitled to a certificate from said board. This board may in its discretion arrange for reciprocity in licenses with the authorities of other States and Territories having requirements equal to those established by this act. Licenses may be granted applicants for licenses under such reciprocity on payment of twenty dollars.

SEC. 9. All applicants to practice the healing art in this State not licensed under the provisions of the previous sections must successfully pass an examination before the Board of Medical Examiners. Applicants to be eligible for examination must present satisfactory evidence to the board that they are more than 21 years of age, of good moral character and graduates of bona fide, reputable medical schools. Application for examination must be made in writing under affidavit to the secretary of the board on forms prepared by the board accompanied by a fee of $15, and such applicants shall receive a notice of the date and place of examination; except when an applicant desires to practice obstetrics alone the fee shall be $5.00. Applicants to practice obstetrics in the State of Texas, upon proper application, shall be examined by the board in obstetrics only, and upon satisfactory examination shall be licensed to practice that special branch, provided this shall not apply to those who do not follow obstetrics as a profession, and who do not advertise themselves as obstetricians or hold themselves out to the public as so practicing. In case any applicant from unsatisfactory examination be refused a license, he or she shall, after one year, be permitted to take a second examination without an additional fee. All certificates shall be attested by the seal and signed by all members of the board.

SEC. 10. The fund realized from the aforesaid fees shall be applied first to the payment of the necessary expenses of the board of examiners, any remaining funds shall be applied by the order of the board to compensating the secretary and members of the board proportional to their labors.

SEC. 11. All examinations shall be conducted in writing and in such manner as shall be entirely fair and impartial, the applicants being known by numbers, without names or other method of identification on examination papers by which members of the board may be able to identify such papers until after applicants have been granted licenses or rejected. Examinations shall be conducted on the scientific branches of the healing art only, and shall include *anatomy, physiology, chemistry, histology, bacteriology, pathology, physical diagnosis, surgery, obstetrics, gynecology, hygiene, and medical jurisprudence.* All questions and answers with grade attached shall be preserved for one year. All applicants examined at the same time shall have the same questions in all branches.

SEC. 12. Nothing in this act shall be so construed as to discriminate against any particular school or system of practice. In case any unforeseen differences of opinion should arise among members of the board belonging to different schools, such differences should be settled by a committee of the board, appointed by its president, in which each school of medicine shall have equal representation. Nothing in this act shall be construed to prohibit gratuitous service in cases of emergency. It shall not apply to trained nurses, masseurs, or to dentists legally qualified and registered under the laws of this State, or to commissioned or contract surgeons in the United States army, navy or Public Health and Marine Hospital Service, or to legally qualified physicians of other States called in consultation, but who do not open offices or appoint places in this State where patients may be met or calls received.

SEC. 13. The State Board of Medical Examiners may refuse persons to its examinations or to issue the certificate provided for in this act for any of the following causes:

First. The presentation to the board of any license, certificate or diploma which was illegally or fraudulently obtained, or when fraud or deception is practiced in passing the examination.

Second. Procuring or aiding or abetting the procuring of a criminal abortion, or conviction of a crime.

Third. Or other grossly unprofessional or dishonorable conduct of a character likely to deceive or defraud the public, or for habits of intemperance or drug addiction calculated to threaten the lives of patients.

SEC. 14. The right herein to practice the healing art in this State may be revoked by any court of competent jurisdiction, upon proof of the violation of law in any respect in regard thereto, and it shall be the duty of the Board of Medical Examiners and of any member thereof to institute such suit in the name of the State upon the relation of such board or any such member. Such action shall be in the nature of a quo warranto and shall be governed as near as practicable by the law and rules relative thereto.

SEC. 15. It shall be the duty of members of the State Board of Medical Examiners, and of the city and county boards of health and county health officers to bring to the attention of the courts any violation of the law within their respective jurisdictions, and it is hereby made the duty of county and district attorneys to prosecute such violators.

SEC. 16. Any person shall be regarded as practicing the healing art within the meaning of this act who shall profess publicly to be a physician or surgeon and shall offer for practice as such by any system or method for those needing medical or surgical aid, and shall charge therefor money or other compensation. This act shall be so construed as to include persons not pretending to be physicians who offer for sale publicly on the streets or other public places remedies which they recommend for the cure of disease.

SEC. 17. Any person practicing the healing art in this State, as defined in this act, in violation of the provisions of this act shall upon conviction be fined not less than fifty dollars nor more than five hundred dollars for each offense or both by fine and imprisonment not exceeding six months in the county jail, and in no case where any provision of this act is violated shall the violator be entitled to recover by action, suit or warrant any compensation for the service rendered.

SEC. 18. All law and parts of law in conflict with this act are hereby repealed on and after September 1, 1907, and it is expressly provided that all certificates issued by the board under former laws are hereby confirmed and in force, and are hereby made as subject to the provisions of this act as though issued under it.

